# Notes and News

**Published:** 1904-02-27

**Authors:** 


### Notes and News.

Under the will of the late Mr. William Lee, of Manchester, £200 each is left to the Salford Royal Hospital, St. Mary's Hospital, Manchester, and the Children's Hospital, Pendlebury.

At the annual meeting of the Royal Southern Hospital, Liverpool, which was held last week, it was reported that the tropical ward last year had 149 in-patients, as compared with 137 in the previous year. The diseases treated included malaria, dysentery, beriberi, blackwater fever, and sleeping sickness, a large number of different nationalities being represented among the patients. The committee expressed the belief that the institution of a diploma in tropical medicine, contemplated by Liverpool University, would still further attract students to their school.

It is intimated that steps are being taken to introduce more generally into the army a system of training to improve the soldier's power of observation and to develop his quickness of perception, in order that he may be able, under ordinary conditions, to find his way about, and locate an enemy without his presence being disclosed; also to acquire an eye for country which will enable him to report accurately what he has seen, and to instinctively select the position which will afford the best cover and best field of fire, as well as the most suitable line of advance and retreat.

Speaking at a luncheon on Monday, given by the President of the Liverpool Chamber of Commerce, Professor Boyce, of Liverpool University, who has just returned from Egypt, where he has been on a short visit in order to inquire into the result of the anti-malarial expedition—which was sent about 18 months ago to Ismailia by the Liverpool School of Tropical Medicine—stated that as a result of the campaign against the malaria-bearing mosquito, the average number of cases of malaria had fallen from something like 2,000 per annum to 200. There had been no deaths amongst Europeans during the past year, and only four amongst the natives, as against 30 deaths in the previous year.

The Queen and the Prince and Princess of Wales have given their patronage to a concert, organised by Signorina Guilia Ravogli, and fixed for Friday, June 3, at the Queen's Hall, in aid of the Appeal Fund of St. Bartholomew's Hospital.

At the annual meeting of the Manchester Children's Hospital, Pendlebury, last Friday, it was announced that in consequence of the increase of expenses over receipts, it had been found necessary to transfer £2,000 from the capital account to the income account.

In opening the new workhouse infirmary of the Holsworthy Union a few days ago, the Local Government Board Inspector, Mr. H. Preston Thomas, declared that it was "one of the best and most compact that he knew." The building, which cost £1,500 to erect and furnish, contains four bedrooms, two sick wards with six beds in each, lying-in ward on ground floor, nurses' bedrooms, and bath-rooms.

An appeal is being issued on behalf of Hampton's Mission for the Blind Poor for the sum of £4,500, which is required to erect a new Mission Hall and Home of Rest for young blind women workers. A site has already been secured in the Borough Road, S.E., and it is intended to commence building at once. It is stated that £25 will provide a cubicle for a blind woman, while two guineas will furnish a cubicle. Donations should be sent to Lord Llangattock, at the Temporary Mission Hall, Westminster Bridge Road, S.E.

The committee of the King's College Hospital Removal Fund have taken Olympia provisionally for a few days in May, with the intention of reviving the famous Eglinton Tournament of 1839. The tournament lasted for three days at Eglinton Castle, in Ayrshire, and was on a most elaborate scale, though to some extent it was marred on the first two days by bad weather. After the knights had finished tilting with broomsticks, Lord Eglinton made a speech in which he remarked that he was now convinced that "the spirit of chivalry had not slept too long to be awakened."

The death of Dr. William Macleod, C.B., Inspector-General of Hospitals and Fleets, Royal Navy, retired, is announced at the age of 84. Entering the Navy in 1842 as an assistant surgeon, Dr. Macleod served on the China Station in that rank from 1845 to 1847. He was surgeon of the *Driver* in the Baltic during the American War, and of the *Madagascar*, in 1859, at Rio de Janeiro, during a severe epidemic of yellow fever. In 1866 he was promoted to the rank of Deputy-Inspector-General, and had charge of the Royal Naval Hospital at Yarmouth from that year until April 1875. He was then made Inspector-General of Hospitals and Fleets, continuing his charge at Yarmouth until 1880, when he retired from the Navy with a C.B. Eight years later he received a Greenwich Hospital pension in further recognition of his services. He was the author of several papers on medical subjects.

